# Identification and Analysis of Conserved *cis*-Regulatory Regions of the *MEIS1* Gene

**DOI:** 10.1371/journal.pone.0033617

**Published:** 2012-03-20

**Authors:** José Luis Royo, José Bessa, Carmen Hidalgo, Ana Fernández-Miñán, Juan J. Tena, Yolanda Roncero, José Luis Gómez-Skarmeta, Fernando Casares

**Affiliations:** Centro Andaluz de Biología del Desarrollo (CABD) CSIC-UPO-Junta de Anadalucía, Sevilla, Spain; The University of Chicago, United States of America

## Abstract

Meis1, a conserved transcription factor of the TALE-homeodomain class, is expressed in a wide variety of tissues during development. Its complex expression pattern is likely to be controlled by an equally complex regulatory landscape. Here we have scanned the Meis1 locus for regulatory elements and found 13 non-coding regions, highly conserved between humans and teleost fishes, that have enhancer activity in stable transgenic zebrafish lines. All these regions are syntenic in most vertebrates. The composite expression of all these enhancer elements recapitulate most of Meis1 expression during early embryogenesis, indicating they comprise a basic set of regulatory elements of the Meis1 gene. Using bioinformatic tools, we identify a number of potential binding sites for transcription factors that are compatible with the regulation of these enhancers. Specifically, HHc2:066650, which is expressed in the developing retina and optic tectum, harbors several predicted Pax6 sites. Biochemical, functional and transgenic assays indicate that *pax6* genes directly regulate HHc2:066650 activity.

## Introduction

Meis genes belong to the TALE-homeodomain class of conserved transcription factors. Together with their *Drosophila* homologue, *homothorax (hth)*, they are known to be required for the development of many organs in vertebrates and invertebrates [1], [2], [3]. Molecularly, Meis proteins are known partners of PBX family proteins, also TALE-homeodomain transcription factors and, together with them, interact with region and organ-specific transcription factors, such as Hox, MyoD or Pax proteins [2], [4], [5], [6]. The founding member of the family in vertebrates, Meis1 (myeloid ecotropic insertion site 1, [7]), has also been found miss-expressed in a number of tumor types, including acute myeloid leukemia [8], lung adenocarcinoma tumors [9], neuroblastomas [10], [11], [12], ovarian carcinomas [13] and nephroblastomas [14].

Part of the pleiotropic developmental roles of Meis genes reside in their complex and dynamic expression patterns. In zebrafish, early *meis1* expression is detected in the developing eye, and in the midbrain and hindbrain regions. Later on, *meis1* expression is found in the ciliary marginal zone of the eye, optic tectum, forebrain (presumably olfactory bulb) and branchial arches [15], [16]. In other vertebrates, Meis1 has been also detected in the somites and proximal fore- and hind-limbs at different developmental stages, as well as in the developing haematopoietic system and the pancreas [17], [18], [19]. This complex expression profile is likely to be controlled by an equally complex regulatory landscape. However, very few studies have been carried out on the transcriptional regulation of Meis genes [20]. In order to start unraveling this complex regulation, we have set to identify *cis*-regulatory elements (CREs) of the *meis1* gene. First, we have used a criterion of synteny and evolutionary conservation [21] to extract non-coding DNA sequences from the *meis1* locus conserved from human to fish. This approach has been shown to be useful in CRE identification [22]. Second, we have tested the enhancer potential of these human sequences *in vivo* using zebrafish transgenesis. This analysis has allowed us to find CREs containing transcriptional enhancer activity with different degrees of tissue-specificity. Finally, we have tried to identify potential *trans*-regulators of the identified *meis1* enhancers, using bioinformatics and gene expression data information. In all, this paper presents a first attempt at unraveling the transcriptional regulatory complexity of the vertebrate Meis1 locus, identifying a number of tissue-specific CREs and predicting new potential regulators of Meis1 CREs.

## Materials and Methods

### Animal care

Zebrafish (*Danio rerio*) were maintained in our breeding colony according to standard procedures (http://zfin.org). Embryos used for Tol2-mediated transgenesis were obtained from the wild-type AB/Tuebingen (AB/TU) zebrafish strain. Potential transgenic founders were out-crossed to a TUP strain. Fertilized eggs were kept at 28°C in E3 medium with 0.003% 1-phenyl-2-thiourea to prevent pigmentation and were staged according to Kimmel et al. [23].

### Candidate region selection criteria

Human sequences (genome release: hg18) were extracted from those with at least 75% identity to mouse (genome release: mm8) and at least 100 bp long. We removed the human-mouse conserved elements that overlapped with gene annotations, human mRNAs or repeats. The remaining human-mouse conserved non-coding elements were clustered if they were 3 Kb or closer from each other. We then checked if the clustered region had human-chicken (genome release: galGal2), human-frog (genome release: xenTro2) and human-zebrafish (genome release: danRer5) conservation (at least 70% identity to human and at least 50 bp long). In order to call a region as highly conserved non-coding region (HCNR), we demanded that the clustered human-mouse region overlapped with the respective conserved elements from all the other species (chicken, frog and zebrafish). We extended the HCNR 150 bp on both sides and looked for primers between 18 and 27 bp in those flanking regions (Table 1).

**Table 1:** Human highly conserved non-coding regions assayed for enhancer activity.

Region (Hg18)	Construct name	G_0_ GFP activity
chr2:65915131–65916370	HHc2:065915	YES
chr2:65944793–65946456	HHc2:065944	YES
chr2:65946418–65947702	HHc2:066515	n.d.
chr2:66363945–66365004	HHc2:066523	n.d.
chr2:66515157–66516033	HHc2:066543	YES
chr2:66523076–66524718	HHc2:066588	YES
chr2:66541547–66543530	HHc2:066589	YES
chr2:66543660–66544441	HHc2:066603	YES
chr2:66549771–66550550	HHc2:066617	YES
chr2:66588227–66589754	HHc2:066628	YES
chr2:66589737–66591000	HHc2:066629	n.d.
chr2:66628254–66628945	HHc2:066650	YES
chr2:66635244–66637062	HHc2:066659	YES
chr2:67361651–67363128	HHc2:066769	n.d.
chr2:66649442–66651672	HHc2:066775	YES
chr2:66651589–66653505	HHc2:067135	YES
chr2:66654727–66656625	HHc2:067156	YES
chr2:66658870–66660479	HHc2:067347	YES
chr2:66768926–66770178	HHc2:067361	YES
chr2:66775251–66777554	HHc2:067641	YES
chr2:67075335–67076935	HHc2:066104	YES
chr2:67134578–67136311	HHc2:066522	YES
chr2:67155556–67157246	HHc2:066569	YES
chr2:67316077–67317518	HHc2:066644	YES
chr2:67347336–67349242	HHc2:066683	YES
chr2:67361651–67363128	HHc2:066719	n.d.
chr2:67398590–67400452	HHc2:066758	n.d.
chr2:67641486–67643007	HHc2:066780	YES
chr2:67934055–67936018	HHc2:067044	n.d.
chr2:66103519–66104070	HHc2:067086	n.d.
chr2:66264836–66265369	HHc2:067288	n.d.
chr2:66442170–66442486	HHc2:067353	n.d.
chr2:66443231–66443891	HHc2:067289	n.d.
chr2:66522162–66522381	HHc2:067353	n.d.

Annotation of the regions corresponding to the human Hg18, that were amplified by PCR and assayed for enhancer activity using the ZED vector. (n.d: not detected).

### ZED-HCNR Collection

Human HCNR fragments where PCR-amplified using HiFi Taq polymerase (Roche, Manheim, Germany) using standard procedures. PCR products were cloned into the pCR8/GW/TOPO vector (Invitrogen, Pasadena, USA). HCNR-containing clones were recombined into the Zebrafish Enhancer Detection (ZED) shuttle transgenesis vector [24]. Briefly, ZED contains two modules flanked by Medaka (*Oryza latipes*) TOL2 transposase target sites, enabling efficient transgenesis, as previously described [25]. The first module contains the minimal GATA promoter driving the expression of the enhanced green fluorescent protein (GFP). All HCNRs were cloned upstream of this module using the Gateway system (Invitrogen, Pasadena, USA). Two strong insulators, which reduce the potential influence of the regulatory elements that may be present in the vicinity of the integration sites, flank this reporter cassette. The second module contains the cardiac actin promoter driving the expression of the red fluorescent protein (RFP), which serves as a positive control for transgenesis in F0 and F1 embryos [24].

### Selection of enhancer-containing HCNRs candidates

One-cell stage embryos were injected with 3–5 nl of a solution containing 25 ng/µl of each construct and 25 ng/µl of TOL2 mRNA. A minimum of 300 embryos were injected per experiment. Embryos where then incubated at 28°C as previously described. GFP expression was evaluated 24, 48 and 72 hours post-fertilization. Whenever GFP was observed, the HCNR tested was considered as a potential candidate and embryos were selected and raised to sexual maturity to be analyzed in F1. Both somites and heart expression of RFP were used as positive control for transgenesis and served to discard any HCNR without enhancer activity. For high-resolution pictures a F-View black/white digital camera coupled to a WD70 Nikon camera was used. Adobe Photoshop was used to adjust bright and contrast.

### Enhancer mutational assays

Mutation of the potential Pax6 binding sites was performed using site-directed PCR-based mutagenesis over the pCR8 TOPO containing the HHc2:066650 HCNR using the following mutagenic oligos (5′-3′): Mut1F: CATAAATCTGTCTAACCGCACATTT CTACACTAACCTGC, Mut1R: GCAGGTTAGTGTAGAAATGTGCGGTTAGAC AGATTTATG and Mut2F: CTTGCTTGTGTCTCTGATTATAAAAATCACTGCT GAGGCC, Mut2R: GGCCTCAGCAGTGATTTTTATAATCAGAGACACAAG CAAG. The resulting construct was recombined into the ZED vector. Upon injection, mosaic embryos were selected according to the presence of RFP expression in the somites, which served as an injection control. This selection was made double blind by a different operator. Embryos were then dechorionated and photographed using identical exposures. The eye GFP expression for each embryo was normalized with its somite RFP expression.

### Whole-mount *in situ* hybridization

Embryos were raised at 28°C until fixed in 4% paraformaldehyde in PBS. Whole-mount *in situ* hybridization was performed as previously described [26] using a digoxigenin-labeled anti-*gfp* probe. For staining, anti-digoxigenin, an alkaline-phosphatase conjugated antibody followed by NBT/BCIP was used. *meis1* ISH was performed as previously described [27].

### Chromatin immunoprecipitation

For immunopreciptation assays we used a c-myc-tagged Pax6 protein, produced by fusing a c-Myc epitope to the 5′ end of *Oryza latipes* Pax6 full length open reading frame (Genebank accession number CAA04395.1) (L. Beccari and P. Bovolenta, unpublished results). One-cell stage embryos were injected with 100 pg of a Pax6::Myc capped mRNA and incubated for 24 hpf before fixation with 1.85% formaldehyde. Nuclei were further isolated prior to sonication using Bioruptor (Diagenoder, Belgium). Lysates were incubated with monoclonal anti-Myc (9E10, Babco#MMS150R-500) conjugated with Magnetic Protein-G Dynabeads (Invitrogen, Norway). DNA was purified from both input lysates and immunoprecipitated samples and analyzed using using real time qPCR. Oligos used were zHHc2:066650F: TGCCTTTGCCATTAGTAATCC and zHHc2:066650R: CAGCCAACACATAGCCACAC. Amplifications were normalized according to a random region from the zebrafish genome ControlF: AACAGCTACCGGTAATAAACT and ControlR: AGGAAACACTGCCAAATAA GC.

### Chromatin conformation capture (3C) assays

3C assays were performed according to standard procedures using whole zebrafish embryos at 24 hpf [28]. Zebrafish BAC CH211-216I21 spanning 214 kb covering the region of interest, was used for normalization. Oligos were designed using Primer3 software pointing towards the selected *Hind*III genomic restriction sites. Downstream 3C_control: GGCTTAGCATCTTGTCAATGC, Upstream 3C_control: TCGAAGACAACTGTCAGCTTTG, the zHHc2:066650: TAACATGGCCAGAAATG TGC, promoter anchor: AATGCCACTATCACTGCAAATG.

### Web resources

For representation and non-coding conservation analysis of the *meis1* locus, we used the VISTA browser, taking as a reference the human hg18 genome release [29] (http://pipeline.lbl.gov/cgi-bin/gateway2). Synteny blocks were extracted from http://ecrbrowser.dcode.org/ [30]. For comparative potential transcription factor binding sites, the Consite Web Resource was used, using the positional weight matrixes loaded from JASPAR [31] (http://asp.ii.uib.no:8090/cgi-bin/CONSITE/consite/). Annotated expression patterns of different transcription factors were obtained from ZFIN (http://zfin.org).

## Results

### *meis1* HCNRs are enriched in tissue-specific enhancers

Analysis of the genomic region surrounding the human *meis1* transcriptional unit identifies a genomic desert of ≈1.5 Mb on chromosome 2 between *SPRED2* and *ETAA1* genes. This region has preserved its syntenic organization since the last common ancestor of teleost fishes and humans (Figure S1), suggesting that the large non-coding region surrounding the *meis1* transcriptional unit have been maintained as a large regulatory domain. Although this vast area represents just ≈0.7% of human chromosome 2, it contains 18% of all HCNRs present on this chromosome. This HCNR enrichment has been previously shown to be characteristic of developmental genes, and has been suggested to be associated to their complex spatiotemporal expression patterns [22], [32]–[36]. Using our own selection criteria (see methods for a detailed description) together with the visual inspection of previously annotated HCNRs via the VISTA browser [37], [38], we selected a total of 34 HCNRs present in the human *meis1* locus (Hg18, chr2:065.893.545–067.353.996) that exhibited a high degree of conservation among vertebrates. These regions were amplified from human DNA and assayed for enhancer activity using transgenesis in zebrafish. Upon an initial evaluation at 24 and 48 hours post fertilization (hpf) in injected (mosaic) embryos, we detected GFP activity in 65% (22 out of 34; Table 1) of the assayed HCNRs, a surprisingly high proportion of the sequences tested. Mosaic embryos injected with these constructs were raised to sexual maturity and their progeny screened for stable transgenic lines.

**Figure 1:**
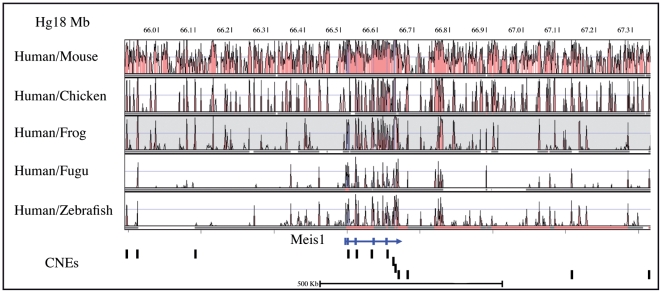
Distribution of the human conserved non-coding regions showing enhancer activity in transgenic Zebrafish. Vista browser representation of the genomic region surrounding the human *MEIS1* gene (chr2:065.893.545–067.353.996), using Hg18 human genome as reference. Conservation is represented as pink peaks. Conserved non-coding elements (CNEs) with confirmed enhancer activity in stable transgenic zebrafish lines are marked as vertical bars.

**Figure 2:**
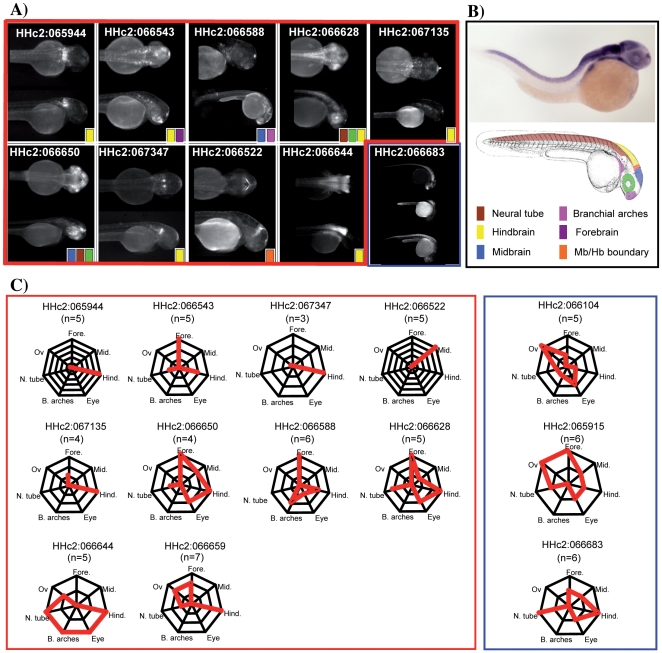
Enhancer activities from the HCNRs recapitulate endogenous *meis1* expression pattern. A) Dorsal and lateral views of a representative founder illustrate the expression pattern of each HCNR. B) Picture of a *meis1 in situ* hybridization of a 30 hpf embryo illustrates the different expression territories. A color-based schema of a zebrafish embryo highlights the different domains where GFP expression was found during the study. C) Diagrams of patterns of expressions of highly penetrant enhancers (boxed in red) and of non-tissue specific enhancers (boxed in blue). In each diagram, the number of founder lines showing expression in each particular body structure is represented by the red lines. For example, of three lines of HHc2:066543, all three showed forebrain expression, and additionally, two of them drove expression in the hindbrain while the remaining one in the neural tube. *In situ* hybridization picture is from ZFIN [16].

Upon outcrossing, we found reporter cassette transmission in offsprings for 19 HCNRs, clearly detected by the somites-specific expression of the transgenesis reporter control (RFP). GFP expression patterns of stable transgenic lines are summarized in table 2. Thirteen HCNRs out of the nineteen (68%) exhibited clear enhancer activity when compared to stable lines carrying just the empty backbone (Figure 1) [39], [40]. Among them, 9 HCNRs exhibited tissue-specific enhancer activity, consistently shared by all the lines (no. of founders ≥3) established for each of them, as illustrated in Figure 2 (for a full annotation see Figure S2). As previously described, *meis1* is expressed in the segmental plate, midbrain, hindbrain and the eye field at 12 hpf. At 24 hpf *meis1* can be also detected in the somites and the spinal chord while the expression in the hindbrain turns more intense in the rhombomeres. At this stage *meis1* eye expression is restricted to the retina, the expression in the somites decays while appearing in novel territories such as the olfactory bulb and the optic tectum. Finally, at 48 hpf, branchial arches and pronephric ducts also start expressing *meis1* [16]. As illustrated in Figure 2, several HCNRs drive the expression to different territories of the central nervous system. HHc2:065944 shows expression in the rhombomeres, while HHc2:066543 show expression in the telencephalon (presumable olfactory bulb). Both HHc2:066628 and HHc2:066644 drive the expression of the GFP to the spinal chord. HHc2:066543 together with HHc2:067135 exhibit also weak expression in some particular hindbrain cells. The overlap between *meis1* endogenous pattern and those driven by the different HCNRs was particularly evident for HHc2:066650 (retina, tectum and neural crest derivatives) and HHc2:066644 (hindbrain, neural tube and branchial arches). Therefore, the HCNRs with enhancer activity are expressed in domains of the expression pattern of *meis1* (Figure 2, panel B), except in somites, as no somite-specific enhancers were detected among the different HCNRs assayed. The only exception to this general rule was observed for HHc2:066522, which drives GFP expression to the midbrain/hindbrain boundary, a territory where *meis1* is not expressed. This ectopic expression could be explained if some important regulatory sequences were not included in the cloned fragment.

**Table 2:** Enhancer activity displayed by the HCNRs in stable transgenesis at 24–48 hpf.

Construct	Founders (n)	Enhancer activity in F_1_	Forebrain	Midbrain	Hindbrain	Eye	Branchial arches	Neural tube	Otic vesicle	Notes
HHc2:065915	6	+	2	1	1	2	-	1	2	Booster: Larynx, lens and hatching gland
HHc2:065944	5	+++	-	-	5	-	-	1	-	When hindbrain also Rhombomere
HHc2:066541	5	−	-	-	-	1	-	-	1	
HHc2:066543	5	+	3	-	2	-	-	1	2	
HHc2:066588	6	+++	3	-	2	1	2	-	1	Notochord, n = 1
HHc2:066628	5	++	3	1	4	3	-	2	-	Fins: n = 1
HHc2:066650	4	+++	3	2	3	2	-	1	-	
HHc2:066659	7	+++	2	-	3	-	-	1	2	
HHc2:066775	3	−	-	-	-	-	-	-	-	
HHc2:067135	4	+	1	-	3	-	-	-	-	
HHc2:067347	3	+++	1	-	3	-	-	-	-	
HHc2:067361	3	−	-	-	-	-	-	-	-	
HHc2:067641	2	−	-	-	-	-	-	-	-	
HHc2:066104	5	++	1	-	1	2	1	1	3	When eye also lens
HHc2:066522	5	+++	-	4	-	-	-	-	-	Midbrain/hindbrain boundary
HHc2:066569	4	−	-	-	-	-	-	.	-	
HHc2:066644	5	+	-	-	2	-	1	2	1	
HHc2:066683	6	+++	1	1	2	1	-	2	-	Muscles at 24 hpf; n = 1
HHc2:066780	3	−	-	-	-	-	-	-	-	

Table summarizing the GFP expression patterns and the enhancer activity found in the study. The “+” symbol in the enhancer activity column symbolizes the strength of the expression patterns according to GFP expression levels regardless of the similarity among founders. The “−” symbol represents the absence of activity. Columns 4–11 refer to the number of founders showing GFP expression on each territory. A CNE was classified as “negative” if at least two founder lines did not show any GFP expression. To annotate the expression pattern of any positive CNE, at least three stable transgenic lines were analysed. “Booster” refers to HCNRs that, when in different stable lines, drive expression in very different patterns, as if boosting the position effects.

For other HCNRs, patterns of GFP expression were non-overlapping among the founder lines established for each of them (HHc2:066683, HHc2:066104 or HHc2:065915). This suggested that these HCNRs were still able to recruit basic transcriptional machinery out of their genomic context, but lacked a clear tissue-specific activity. A full description of the patterns obtained can be found in (Figure S2). The remaining 6 HCNRs showed somite RFP expression but no GFP, and were therefore considered false positives. For the remaining constructs no transgene transmission was obtained.

### Exploring potential regulators upstream of *mies1* taking advantage of tissue-specific HCNRs

**Figure 3:**
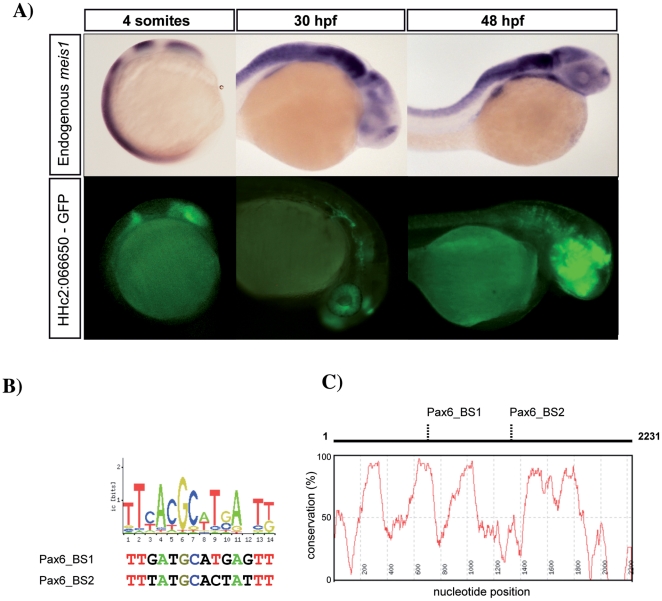
HCNR sequence analysis suggests novel *meis1* upstream regulators. (A) *meis1* transcription, detected by *in situ hybridization* (upper panel) compared to GFP expression, driven by the HHc2:066650-GFP transgenic line (lower panel) at 12 (left), 24 (middle) and 30 hpf (right). Side views are shown. HHc2:066650 drives GFP expression in the eye primordium, the early hindbrain, the retina, the optic tectum and the olfactory bulb, which are *meis1* expression domains. B) The analysis of the potential binding sites predicted by Jaspar highlights the presence of three potential Pax6 sites. Two of these sites contain a high degree of similarity with the consensus. (C) Pax6_Binding_Site1 also mapped to a microisland of ultraconservation when human and zebrafish orthologous regions were aligned.

**Figure 4:**
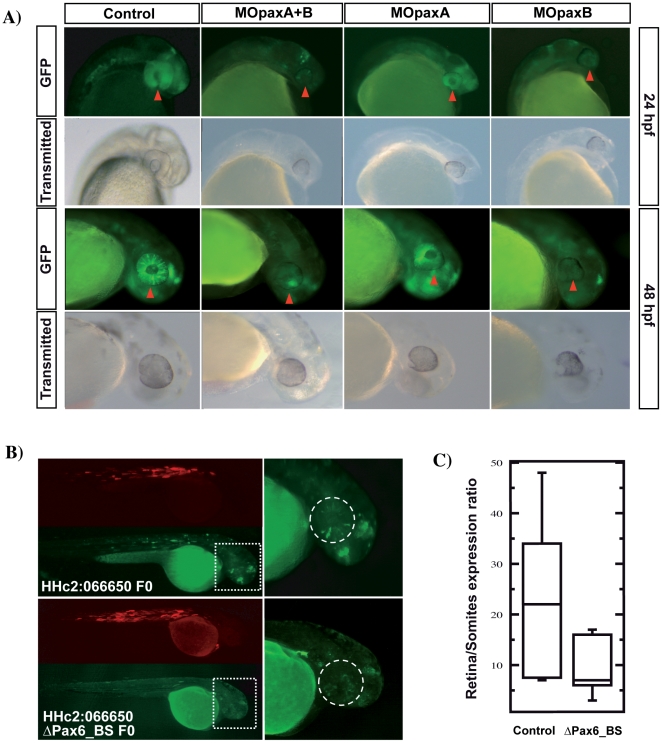
Pax6 regulates the enhancer activity of HHc2:066650. A) Representative pictures of transgenic zebrafish embryos at 24 and 48 hpf, corresponding to HHc2:066650 F2 offspring injected with MOpaxA, MOpaxB, or a mixture of both. The red arrow points to the retina, where a strong decrease in the GFP levels is observed after morpholino treatment. A control mopholino-injected individual is shown for comparison. B) Representative mosaic embryos injected with HHc2:066650 construct with/without the two candidate Pax6 binding sites. Both the GFP and RFP channels are included. The boxed region is magnified on the right. The dashed circle delineates the eye. (C) GFP expression was measured in the eye (area marked by the dashed circle in (B)) of wild type and Δpax6_BS version of HHc2:066650 mosaic embryos (n = 9 and 11 embryos, respectively), and normalized with their respective muscle RFP expression. Enhancer signal significantly decreased in the mutant version of HHc2:066650 (p = 0.018, Mann Whitney test). The box plots contains a central rectangle, which spans from the first quartile to the third quartile. A segment inside the rectangle shows the median and whiskers above and below the box show the locations of the minimum and maximum.

Human HCNR sequences showing enhancer activity were analyzed *in silico* for potential transcription factors binding sites using JASPAR positional weigh matrixes (PWM) at the Consite Web Resource (Table S1). This analysis provided a wide range of potential direct *meis1* upstream regulators. Although extremely sensitive, this approach is particularly prone to detect false positives. To increase the reliability of these predictions, we applied two additional filters. First, we selected those sites with highest PWM scores (in the first quartile). Second, of those, we identified the sites conserved between human and zebrafish, with a conservation cut-off above 75%, according to the analysis carried out using the CONSITE web resource. Then, we analyzed the expression of each of the potential trans-regulators using the *in situ* RNA expression data available at ZFIN (www.zfin.org) and selected those whose expression overlapped with the HCNR-driven GFP expression. Of these potential direct regulators, *runx*1, pbx family members and *pax*6 had been previously shown to interact with *meis*1 [2], [4], [5], [6], although none of these studies provided evidence for them acting as direct upstream regulators of *meis1*. Interestingly, we noted that HCNR HHc2:066650 expressed GFP starting around 12 hpf (Figure 3A), when it is detected in the forming eye vesicles, midbrain and hindbrain, and, in later embryos, in the retina and tectum. This expression pattern coincides with that of *meis1* in the anterior neural tube [15], [16]. Interestingly, Pax6 genes are expressed in the retina and optic tectum in a number of vertebrates [41]–[43]. Moreover, when orthologous sequences from both human and zebrafish were compared, one of the two best-fitting Pax6 potential binding sites (Figure 3D) mapped to the same relative position in the enhancer, within a microisland of ultraconservation inside the HCNR (Figure 3E). To test the requirement of Pax6 for HHc2:066650 enhancer activity in zebrafish, we injected morpholinos against the two zebrafish paralogues, *pax*6a and *pax*6b, alone or in combination, in HHc2:066650-GFP one-cell embryos. As previously described, *pax*6a and *pax*6b morphants showed microphthalmia (Figure S3) [44]. When GFP expression was analyzed in the eye primordia of MO*pax*6a or MO*pax*6b-injected HHc2:066650-GFP embryos, a strong decrease in the number of GFP-positive cells in the retina (normalized for total retinal area) (Figure 4A, Figure S4) was detected. We also noted a decreased expression in the prospective optic tectum. These results indicated that Pax6 gene activity is required for HHc2:066650-enhancer activity.

### Pax6 directly regulates retinal expression of a *meis1* eye enhancer

The results described so far suggested a direct regulation of Pax6 on HHc2:066650. To test this point, we decided to mutate the highest scoring Pax6 binding sites (Pax6_BS1 and 2) and to compare the retinal GFP expression pattern driven by the control and the mutated version of HHc2:066650. For this assay, we took advantage of the high penetrance of the GFP expression driven by HHc2:066650 in F0 (i.e. mosaic) embryos, where nearly 70% of the injected embryos consistently showed a GFP expression pattern comparable to that of the stable transgenic lines (Figure 4B, upper panel). The two constructs (i.e. wild type and ΔPax6_BS HHc2:066650) were injected and GFP signal was quantified. Reporter expression was normalized against RFP somite expression, which gave us a measure of the transformation efficiency in each embryo. As illustrated in Figure 4, the signal in ΔPax6_BS HHc2:066650 was reduced more than two-fold compared to the levels driven by the wild type HCNR (p = 0.018, Mann Whitney test). Even though these results do not rule out the possibility that other Pax6 BSs are active in this enhancer, they indicate that the two mutated BSs are required for normal expression levels.

**Figure 5:**
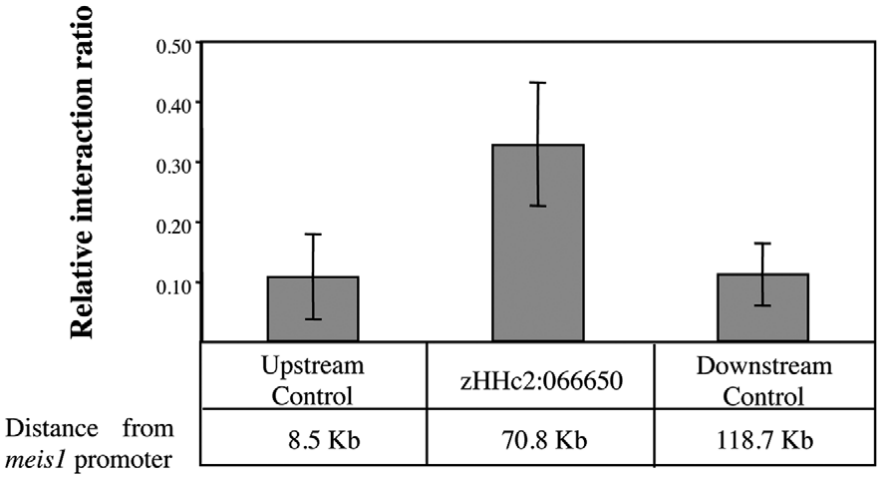
Chromatin conformation assays measuring *in vivo* the relative interaction frequency between zHHc2:066650 and *meis1* promoter. Relative interaction of the zebrafish HHc2:066650 orthologous region and the *meis1* promoter, 70.8 Kb upstream of zHHc2:066650. Interaction frequency was measured relative to two control regions: the first control maps 62 Kb upstream zHHc2:066650, while the second one lies 47.8 Kb downstream the enhancer. Interaction between *meis1* promoter and zHHc2:066650 shows a 3-fold increase when compared to the controls. Error bars represent the standard deviation of three independent measurements.

To test for actual Pax6 binding to HHc2:066650, we injected a Myc-tagged version of Pax6 mRNA in one-celled zebrafish embryos. Chromatin immunoprecipitation using an anti-Myc Ab followed by quantitative amplification of the zebrafish HHc2:066650 orthologous region showed a three-fold enrichment of this CRE relative to input chromatin (Figure S5), supporting direct binding of Pax6 to HHc2:066650. The pattern of expression driven by HHc2:066650 is reminiscent of a subset of pattern elements of the *meis1* gene, strongly suggesting that it is indeed a *meis1* CRE. In order test whether zebrafish HHc2:066650 contacted the endogenous meis1 promoter, we designed a series of 3C experiments. In these assays, the normalized 3C signal of zebrafish HHc2:066650 was significantly higher than that of two control regions mapping proximal and distal, respectively, to it (Figure 5), which demonstrates an *in vivo* physical interaction between this CRE and the *meis1* promoter.

All results described in this section indicated that *pax6* exerted a direct regulation on a *meis1* CRE. In order to test the relevance of this *pax6-meis1* interaction, we asked what effects the manipulation *pax6* levels would have on *meis1* transcription. We evaluated the effects on *meis1* transcription by whole mount *in situ* hybridization in *pax6a*, *pax6b* and *pax6a*+*pax6b* morphant embryos at 18 hpf, but detected no obvious alterations in neither *meis1* pattern nor levels.

## Discussion

Here we present the first systematic scan for conserved *cis*-regulatory regions governing the expression of the *meis1* gene in any vertebrate. Our functional analysis of the HCNRs found in the *meis1* locus reveals that a large proportion of these sequences contain tissue-specific enhancers. These are active in many of the tissues where *meis1* itself is expressed at the stages analyzed, including regions of the neural tube, retina, olfactory bulb and branchial arches. Of note is that, even at these early stages, *meis1* expression in the neural tube is composite, likely resulting from the integration of many independent enhancers. It is also likely that our screening method missed some HCNRs with enhancer activity in small expression domains, since HCNRs were pre-screened for GFP expression in F0. This is because of the mosaic nature of F0 expression, by which large expression domains are more likely to be detected than small ones. This, together with the fact that enhancers can also be found in non-coding DNA that is not evolutionarily conserved [45], [46] suggests that unraveling the upstream regulatory cascades controlling *meis1* expression will be complicated due to the multiplicity of *cis*-regulatory elements. However, the degree of concordance between the enhancer expression territories found in our study and the endogenous *meis1* expression suggests that our analysis has identified a significant number of *meis1* tissue-specific enhancers. In addition, synteny has been found in the gene desert surrounding *meis1* and these non-coding regions show a high degree of conservation across evolution. Altogether, this data allow us to propose that orthologous sequences of these enhancers may also be *meis1* enhancers with similar activity in other vertebrates. Thereby, these HCNRs might comprise an evolutionarily conserved set of *meis1 cis*-regulatory regions.

From the regulatory point of view, we must highlight the interesting behavior found among the different enhancer activities here reported. We observe HCNRs with different degrees of enhancer penetrance, defined as the degree of similarity between the different founders of the same HCNR. Some sequences drive highly reproducible expression patterns (i.e. HHc2:065944, HHc2:067347 or HHc2:066522) while others exhibit some degree of variation in the GFP-expressing territories among founders. This phenomenon could be due to position effects –i.e. the regulatory influence exerted by regulatory regions in the vicinity of the transgene's insertion point. However, we cannot exclude that this variability in expression patterns is attributable to some special properties of these sequences.

The identification of functional CREs is a first step in the investigation of the gene regulatory networks controlling gene expression. Here, we have used information based on binding site composition and gene transcription to identify a number of potential *meis1 trans*-regulators. We have followed different approaches in order to filter out false positives predicted by bioinformatics. Thus, we considered only those transcription factors with the top-scoring PWMs and whose expression overlapped the enhancers' activity at the stages analyzed. Interestingly, some candidates with previously described interaction with *meis1* were found, such as PBXs or Pax6 genes. To date, Pax6 has been shown to be directly regulated by Meis genes during the development of the eye lens and pancreatic islet cells in the mouse [2], [19]. However, a reciprocal regulation of Pax6 on *meis1* has not been described in vertebrates. Under this scenario, we investigated the regulatory relationship between Pax6 genes and the *meis1* retinal and optic tectum enhancer HHc2:066650 in zebrafish. Both morpholino analyses, site-directed mutagenesis of HHc2:066650 and *in vivo* binding assays led us to conclude that Pax6 directly regulates HHc2:066650. In addition we show that zebrafish orthologous HHc2:066650 sequence physically interacts with the *meis1* promoter in its natural genomic context, which confirms that this HCNR is indeed a *meis1* regulatory sequence. The fact that we do not detect alterations in *meis1* expression pattern or levels by *in situ* hybridization in *pax6* morphants suggests that its transcriptional regulation is strongly buffered, perhaps by other CREs with similar enhancer activity, and that the individual contribution of HHc2:066650 is minor. Due to the large impact of Pax6 during normal retinal and brain development, it is of great relevance to identify its downstream targets. Our results suggest that *meis1* should also be considered a direct Pax6 target gene in vertebrates.

## Supporting Information

Figure S1 **Human** ***MEIS1*** **gene lies in a syntenic region among vertebrates.** A) Graphical representation of the *MEIS1* regulatory block found according to ECR genome browser which uses MIME algorithm. B) Detailed correspondence between the Hg18 region of interest and their orthologous genomic regions from mouse (mm9), chicken (galga3), frog (xentro), and zebrafish (danrer05).
(TIF)

Figure S2 **GFP-expressing domains displayed by the different founders.** Summary of the expression patterns from the different founders from all positive *cis*-regulatory regions found in the study. Whenever a particular founder exhibit GFP expression in a defined territory, we refer it as a “1” and the cell is highlighted in green. When no GFP expression is found, the corresponding cell remains grey. Forebrain refers to the presumptive olfactory bulb. M/H_bound. stands for Midbrain-Hindbrain boundary, and D.R. ganglia stands for Dorsal Root ganglia.
(TIF)

Figure S3 **Morpholinos against** ***Pax6*** **a and** ***Pax6*** **b affect eye development.** Representative pictures of 48 hpf wild type zebrafish embryos after different MO injection. The microphthalmia observed among morphants (red arrow) confirmed the functionality of the morpholinos. No effects on control morpholino injected animals were detected.
(TIF)

Figure S4 **Effect of Pax6 MOs on the GFP retinal expression levels of HHc2:066650 stable transgenic line.** Whisker plots showing retinal GFP fluorescence was measured in both morphants and controls (n = 13 for controls, n = 8 for MOpaxA; n = 11 for MOpaxB and n = 13 for MOpaxA+B). * : p-value≤0.05 after Mann Whitney test.
(TIF)

Figure S5 **Chromatin immunoprecipitation of a myc-tagged version of Pax6 shows an enrichment in zHHc2:066650 relative abundance.** Levels of zebrafish HHc2:066650, amplified by PCR, in the chromatin immuoprecipitated with an anti-Myc antibody (ChIP) or in the input chromatin (Input), from 24 hpf embryos injected with 100 pg of a pax6-myc capped mRNA. The figure represents the average and the error bars the standard deviation of three independent analyses.
(TIFF)

Table S1 **Transcription factors hits predicted to bind each human MEIS1 enhancer according to JASPAR dataset scoring within the first quartile.**
(DOC)
