# Supplementary figures and images for: Identification and Analysis of Conserved cis-Regulatory Regions of the MEIS1 Gene
Source: PLoS One. 2012 Mar 20;7(3):e33617. doi: 10.1371/journal.pone.0033617 (PMC3308983; doi:10.1371/journal.pone.0033617)

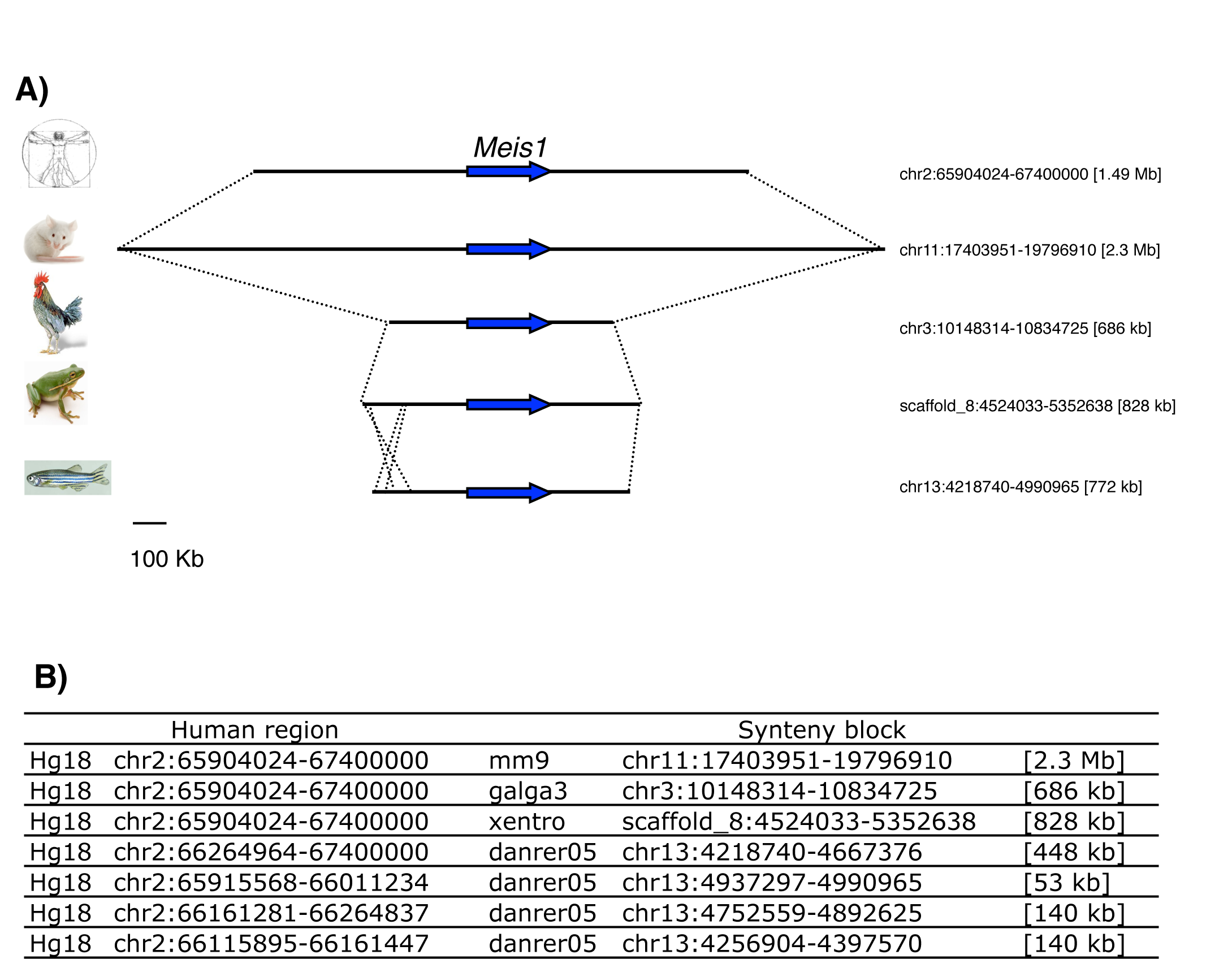

Supplement: Figure S1 — Human MEIS1 gene lies in a syntenic region among vertebrates. A) Graphical representation of the MEIS1 regulatory block found according to ECR genome browser which uses MIME algorithm. B) Detailed correspondence between the Hg18 region of interest and their orthologous genomic regions from mouse (mm9), chicken (galga3), frog (xentro), and zebrafish (danrer05). (TIF) [file pone.0033617.s001.tif]

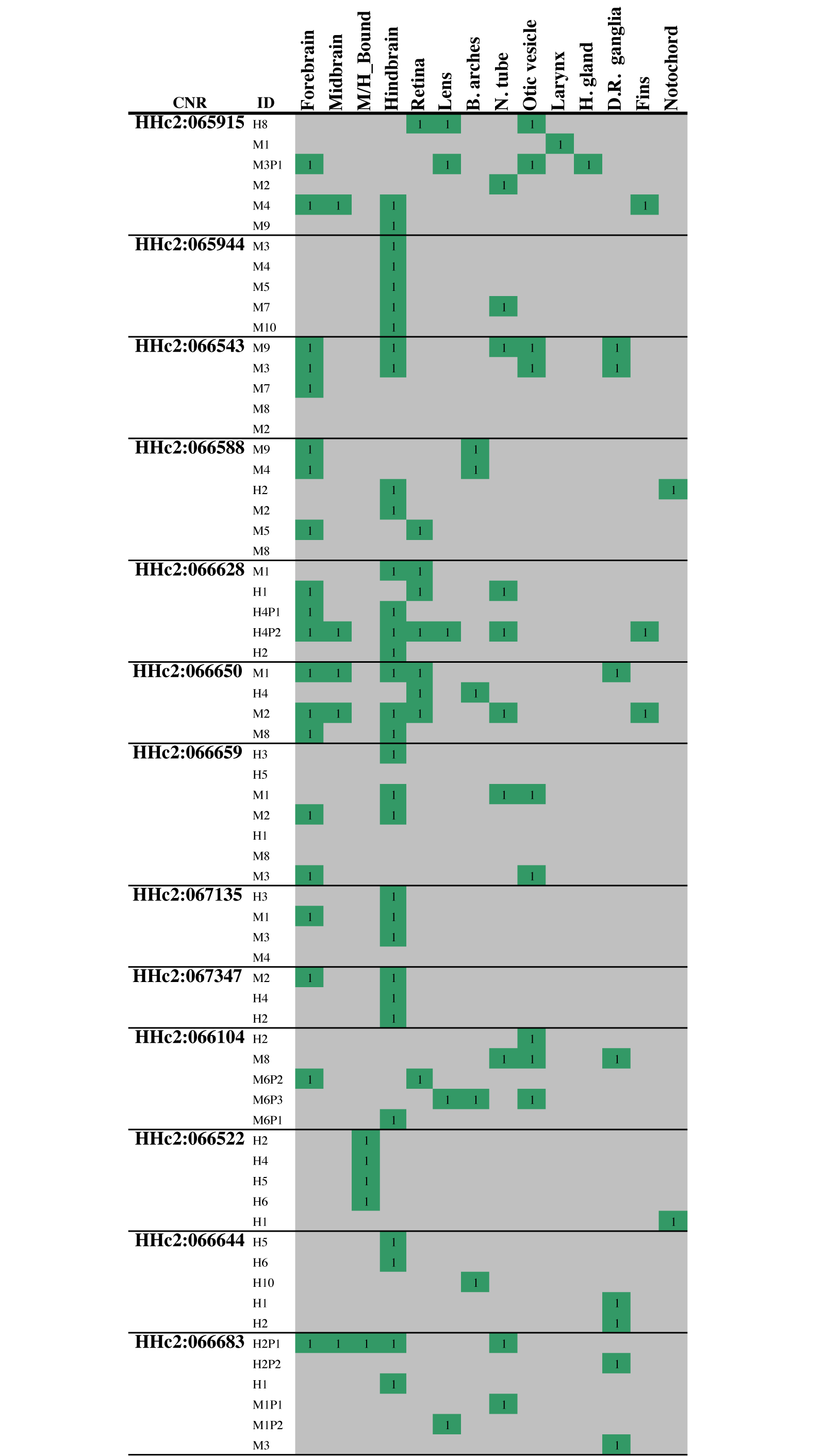

Supplement: Figure S2 — GFP-expressing domains displayed by the different founders. Summary of the expression patterns from the different founders from all positive cis-regulatory regions found in the study. Whenever a particular founder exhibit GFP expression in a defined territory, we refer it as a “1” and the cell is highlighted in green. When no GFP expression is found, the corresponding cell remains grey. Forebrain refers to the presumptive olfactory bulb. M/H_bound. stands for Midbrain-Hindbrain boundary, and D.R. ganglia stands for Dorsal Root ganglia. (TIF) [file pone.0033617.s002.tif]

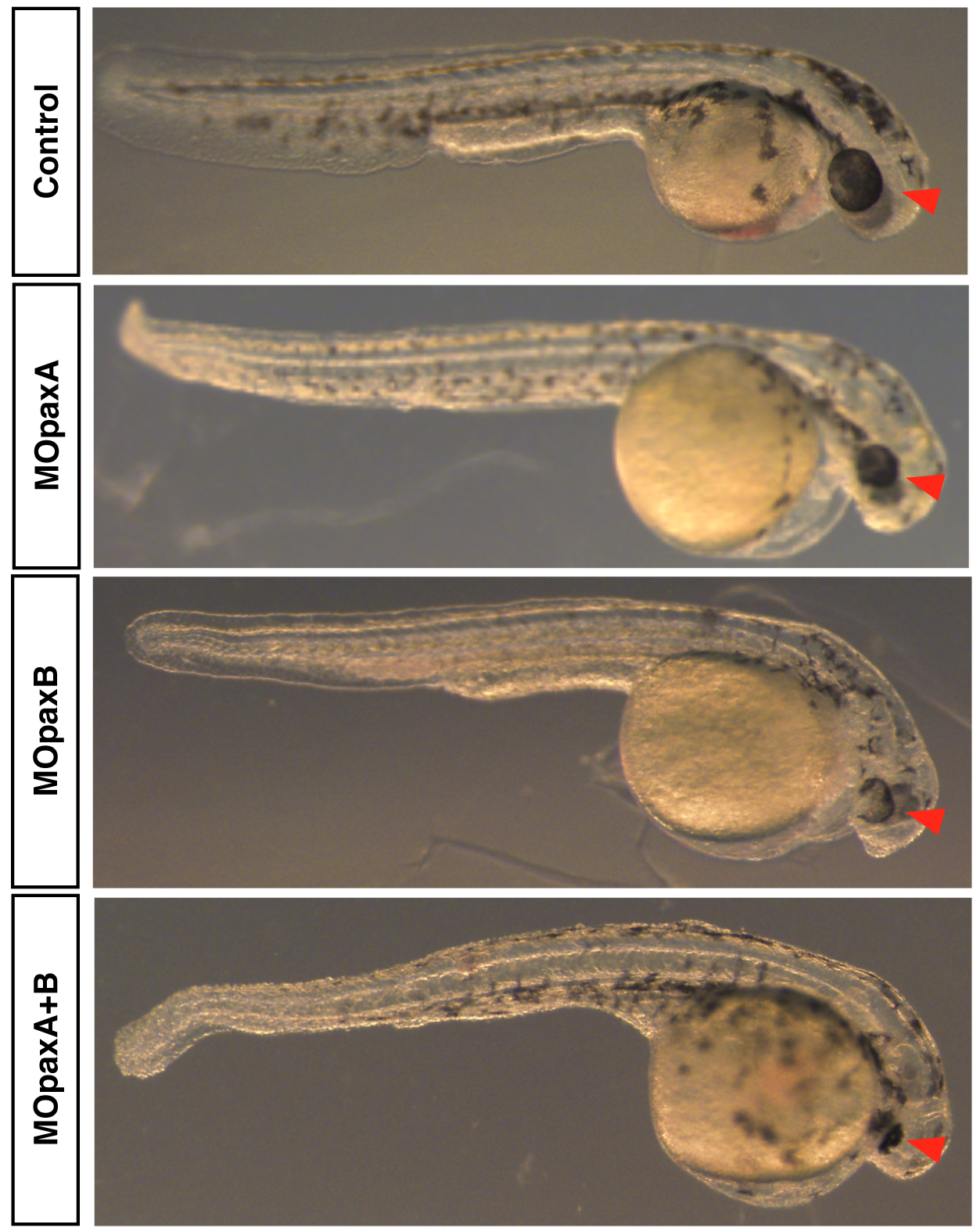

Supplement: Figure S3 — Morpholinos against Pax6 a and Pax6 b affect eye development. Representative pictures of 48 hpf wild type zebrafish embryos after different MO injection. The microphthalmia observed among morphants (red arrow) confirmed the functionality of the morpholinos. No effects on control morpholino injected animals were detected. (TIF) [file pone.0033617.s003.tif]

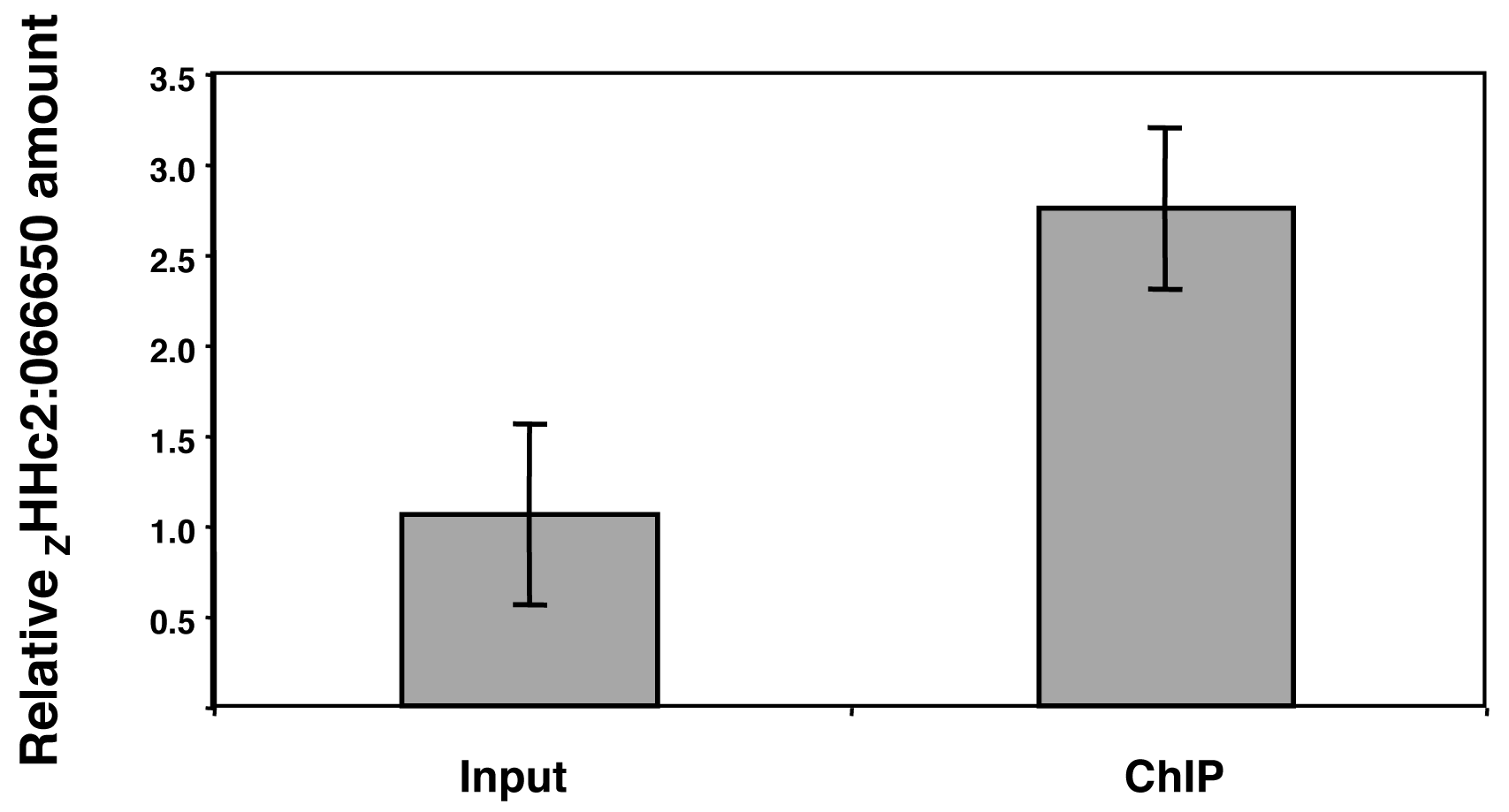

Supplement: Figure S4 — Effect of Pax6 MOs on the GFP retinal expression levels of HHc2:066650 stable transgenic line. Whisker plots showing retinal GFP fluorescence was measured in both morphants and controls (n = 13 for controls, n = 8 for MOpaxA; n = 11 for MOpaxB and n = 13 for MOpaxA+B). * : p-value≤0.05 after Mann Whitney test. (TIF) [file pone.0033617.s004.tif]

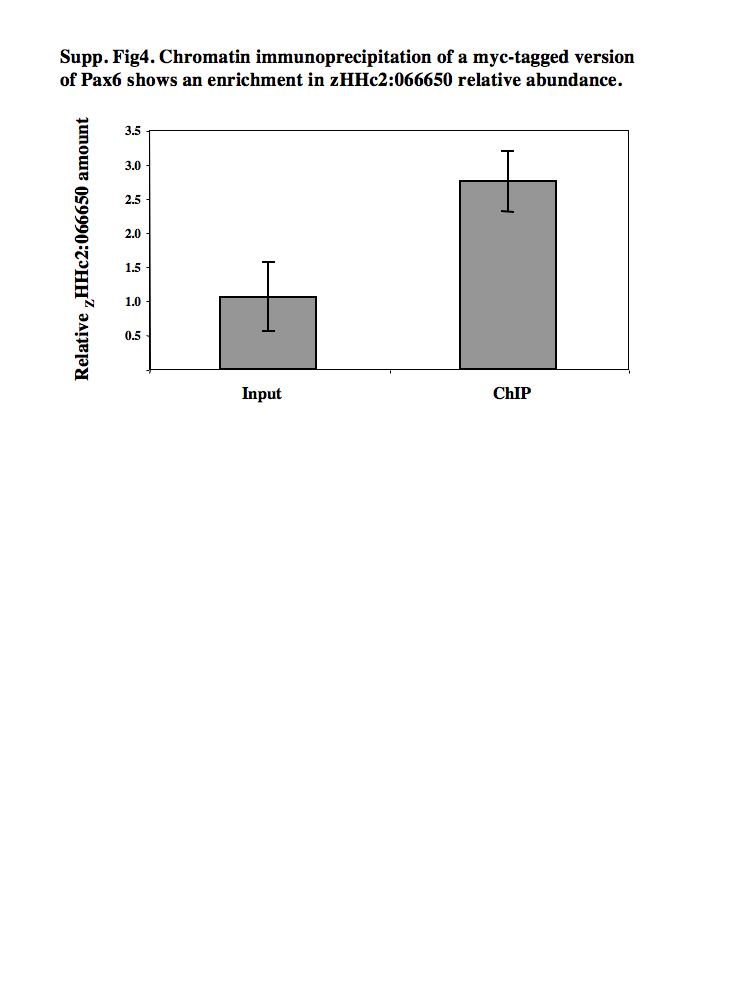

Supplement: Figure S5 — Chromatin immunoprecipitation of a myc-tagged version of Pax6 shows an enrichment in zHHc2:066650 relative abundance. Levels of zebrafish HHc2:066650, amplified by PCR, in the chromatin immuoprecipitated with an anti-Myc antibody (ChIP) or in the input chromatin (Input), from 24 hpf embryos injected with 100 pg of a pax6-myc capped mRNA. The figure represents the average and the error bars the standard deviation of three independent analyses. (TIFF) [file pone.0033617.s005.tif]
