# Supplementary material for: Identification and Analysis of Conserved cis-Regulatory Regions of the MEIS1 Gene
Source: PLoS One. 2012 Mar 20;7(3):e33617. doi: 10.1371/journal.pone.0033617 (PMC3308983; doi:10.1371/journal.pone.0033617)
Supplement: Table S1 — Transcription factors hits predicted to bind each human MEIS1 enhancer according to JASPAR dataset scoring within the first quartile. (DOC) [file pone.0033617.s006.doc]

**Supp. Table I. Transcription factors hits predicted to bind each human MEIS1 enhancer according to JASPAR dataset scoring within the first quartile.**

|  | HHc2:065915 | HHc2:065944 | HHc2:066543 | HHc2:066588 | HHc2:066628 | HHc2:066650 | HHc2:066659 | HHc2:067135 | HHc2:067347 | HHc2:066104 | HHc2:066522 | HHc2:066644 | HHc2:066683 |
| --- | --- | --- | --- | --- | --- | --- | --- | --- | --- | --- | --- | --- | --- |
| AML-1 | 0 | 0 | 1 | 3 | 0 | 3 | 0 | 1 | 1 | 1 | 0 | 0 | 0 |
| Brachyury | 0 | 1 | 0 | 0 | 0 | 1 | 0 | 0 | 0 | 0 | 0 | 0 | 0 |
| C-FOS | 0 | 0 | 2 | 0 | 0 | 1 | 0 | 3 | 0 | 0 | 0 | 0 | 0 |
| CFI-USP | 1 | 1 | 0 | 0 | 0 | 0 | 1 | 1 | 0 | 0 | 0 | 1 | 1 |
| CF2-II | 0 | 3 | 1 | 0 | 2 | 0 | 0 | 2 | 1 | 0 | 0 | 0 | 2 |
| Evi-1 | 0 | 0 | 1 | 0 | 2 | 3 | 0 | 2 | 2 | 2 | 1 | 0 | 0 |
| E4BP4 | 0 | 1 | 0 | 0 | 1 | 1 | 1 | 0 | 1 | 1 | 0 | 2 | 0 |
| FREAC-2 | 2 | 0 | 4 | 2 | 2 | 1 | 0 | 2 | 1 | 0 | 0 | 0 | 1 |
| Hnf-1 | 0 | 2 | 0 | 0 | 1 | 2 | 3 | 2 | 1 | 2 | 0 | 2 | 1 |
| Hnf-3beta | 2 | 5 | 1 | 0 | 3 | 2 | 2 | 3 | 1 | 0 | 0 | 4 | 2 |
| Hunchback (pax1) | 2 | 14 | 1 | 4 | 1 | 7 | 10 | 4 | 4 | 2 | 9 | 2 | 0 |
| irf-1 | 1 | 3 | 0 | 6 | 1 | 6 | 2 | 2 | 2 | 3 | 1 | 2 | 0 |
| MEF2 | 0 | 5 | 1 | 1 | 4 | 2 | 3 | 3 | 2 | 0 | 0 | 1 | 1 |
| Myf | 6 | 1 | 1 | 1 | 1 | 0 | 3 | 2 | 2 | 1 | 1 | 1 | 2 |
| Pax6 | 0 | 1 | 0 | 0 | 0 | 3 | 0 | 0 | 3 | 0 | 0 | 0 | 0 |
| PBX | 1 | 3 | 2 | 0 | 2 | 2 | 1 | 3 | 4 | 1 | 0 | 1 | 0 |
| PPARgamma | 0 | 0 | 0 | 1 | 0 | 0 | 0 | 0 | 0 | 0 | 0 | 0 | 0 |
| P65 | 5 | 3 | 0 | 1 | 0 | 0 | 2 | 1 | 0 | 0 | 1 | 0 | 0 |
| Snail | 1 | 0 | 0 | 1 | 0 | 1 | 0 | 1 | 3 | 0 | 0 | 0 | 0 |
| Sox-5 | 1 | 3 | 2 | 1 | 1 | 7 | 4 | 3 | 4 | 0 | 1 | 3 | 1 |
| Sox17 | 0 | 3 | 0 | 0 | 2 | 1 | 0 | 1 | 2 | 2 | 0 | 2 | 1 |
| Staf | 0 | 1 | 0 | 0 | 0 | 1 | 0 | 2 | 1 | 0 | 1 | 0 | 0 |
| TEF-1 | 3 | 1 | 0 | 0 | 0 | 3 | 0 | 1 | 2 | 0 | 1 | 0 | 1 |
